# Identifying and Responding to Health Misinformation on Reddit Dermatology Forums With Artificially Intelligent Bots Using Natural Language Processing: Design and Evaluation Study

**DOI:** 10.2196/20975

**Published:** 2021-09-30

**Authors:** Monique A Sager, Aditya M Kashyap, Mila Tamminga, Sadhana Ravoori, Christopher Callison-Burch, Jules B Lipoff

**Affiliations:** 1 Perelman School of Medicine University of Pennsylvania Philadelphia, PA United States; 2 Department of Computer Science University of Pennsylvania Philadelphia, PA United States; 3 Department of Dermatology University of Pennsylvania Philadelphia, PA United States

**Keywords:** bots, natural language processing, artificial intelligence, Reddit, medical misinformation, health misinformation, detecting misinformation, dermatology, misinformation

## Abstract

**Background:**

Reddit, the fifth most popular website in the United States, boasts a large and engaged user base on its dermatology forums where users crowdsource free medical opinions*.* Unfortunately, much of the advice provided is unvalidated and could lead to the provision of inappropriate care. Initial testing has revealed that artificially intelligent bots can detect misinformation regarding tanning and essential oils on Reddit dermatology forums and may be able to produce responses to posts containing misinformation.

**Objective:**

To analyze the ability of bots to find and respond to tanning and essential oil–related health misinformation on Reddit’s dermatology forums in a controlled test environment.

**Methods:**

Using natural language processing techniques, we trained bots to target misinformation, using relevant keywords and to post prefabricated responses. By evaluating different model architectures across a held-out test set, we compared performances.

**Results:**

Our models yielded data test accuracies ranging 95%-100%, with a Bidirectional Encoder Representations from Transformers (BERT) fine-tuned model resulting in the highest level of test accuracy. Bots were then able to post corrective prefabricated responses to misinformation in a test environment.

**Conclusions:**

Using a limited data set, bots accurately detected examples of health misinformation within Reddit dermatology forums. Given that these bots can then post prefabricated responses, this technique may allow for interception of misinformation. Providing correct information does not mean that users will be receptive or find such interventions persuasive. Further studies should investigate this strategy’s effectiveness to inform future deployment of bots as a technique in combating health misinformation.

## Introduction

### Background

Health misinformation—defined as information that is incorrect, and possibly intended to deceive [1]—is rampant on the internet. Well-intentioned social media users often advise each other regarding health care treatments and home remedies. Prior studies have assessed health misinformation on Facebook and Twitter, yet one of the most active communities in health care discussions remains less investigated: the social media forums of Reddit [2]. As a social media and commentary platform with 330 million users, Reddit is the fifth most popular site in the United States [3]. The forums, known as subreddits, also cover nearly every medical specialty; for example, dermatology (known as “r/Dermatology”), cardiology, and others.

One of the most active medical forums on Reddit is r/Dermatology, with users seeking to crowdsource for free medical opinions. Indeed, posts often begin with variations of “I cannot afford a dermatologist.” The advice ranges from homeopathic remedies suggested by uncredentialled users to evidence-based medical treatments offered by dermatologists volunteering their time on the forum. A significant portion of medical advice from nonphysicians promotes non–evidence-based homeopathic treatments over scientifically validated medical treatments. For example, a user posting a photograph of a suspicious mole may be falsely reassured by other posters that in-person evaluation is unnecessary or that it can be resolved with the application of essential oils. Given that Reddit posts are anonymous, people may be empowered to reveal their medical concerns more candidly. In contrast, the public nature of a forum such as Facebook may discourage candid sharing [4]. Thus, the design of Reddit makes it a uniquely promising target for studying this crowdsourcing and potential health misinformation.

The audience for Reddit dermatology forums is large and highly engaged; over 55,000 users follow r/Dermatology, over 1.3 million users follow r/SkincareAddiction, and over 19,000 follow r/essentialoils [5]. These users (known as Redditors), log in globally, though a majority are from the United States (58%) [6]. While the majority of Redditors are young men, the skin care forums are largely female-dominated. Subreddit r/SkincareAddiction is one of the largest dermatology-related forums with 87% of female users, of whom 70% are between 19 and 29 years old [7].

### Prior Work

Our previous work has used the artificial intelligence subfield of natural language processing techniques to analyze Reddit dermatology forums’ content [8]. Our data suggest that these forums are a rich source of patient engagement, presenting an untapped opportunity for expert involvement. Our study aimed to investigate the feasibility of engaging in these forums using bots, with the goal of intercepting health misinformation.

Preliminary analysis of Reddit dermatology forums identified a potential target: rampant confusion and misinformation regarding sun exposure. For instance, many users had questions about the dangers of sun exposure, questioning if these supposed dangers are a scam perpetuated by sunscreen companies. Further, tanning beds were often touted as a cure for acne and other skin conditions. Sun exposure–related misinformation was identified as a good target for intervention because of the clear consensus on guidance from the medical establishment. Indoor tanning devices are classified as the highest class of carcinogens by the World Health Organization, and it is well established that tanning bed use is a risk factor for developing melanoma, with multiple tanning bed sessions increasing the risk of melanoma [9-11]. Melanoma leads to an estimated 7000 deaths per year in the United States [12].

Essential oil (EO) use and safety was selected as a second target of misinformation. Users discussed EOs as a remedy for many health conditions, though no such efficacy has been established in the medical literature, and EO use is not without risk. For instance, 1 user solicited information on using EOs to treat Sjogren syndrome and was told to seek out a local herbalist.

In this context, we aimed to develop artificially intelligent bots for Reddit forums as a means to intercept and correct health misinformation.

## Methods

### Methods Overview

To develop bots to intercept health misinformation, we developed 2 sets of machine learning models: 1 targeting posts that discussed sun exposure or tanning, and the second for posts that discussed EOs. We used Google’s BigQuery application programming interface (API) to query publicly available Reddit data [13], pulling from the forums r/Dermatology, r/essentialoils, and r/tanning from January 2018 to August 2019. Google BigQuery API analyses 100% of full-text posts. We used the API to extract Reddit posts and comments that belonged to the subreddits we targeted and then locally ran our scripts over the entirety of the text posts. Using the data from BigQuery, we filtered by subreddit and searched for keywords (Textbox 1) [14].

**Keywords used.**

**Essential oil–related keywords**

- essential oils
- Bay
- cinnamon bark
- clove
- frankincense
- citronella
- cumin
- lemongrass
- lemon verbena
- oregano
- thyme
- lavender
- nutmeg
- peppermint
- tea tree
- cinnamon leaf
- camphor oil
- oil of wintergreen
- jasmine
- ylang-ylang
- sandalwood oil

**Tanning-related keywords**

- sun exposure
- tanning
- tanning bed
- base tan

For the sun exposure/tanning data set, we included all posts from the r/tanning subreddit as positive training instances in addition to posts from r/Dermatology, which contained tanning-related keywords. The remaining posts from r/Dermatology were taken as negative training instances. Similarly, for the essential oils data set, all posts from the r/essentialoils subreddit were considered positive training instances in addition to posts from r/Dermatology that contained EO-related keywords. Positive training instances meant that the targeted content was identified, while negative training instances indicated that no such content was identified within the post. Next, we removed the search keywords from the positive comments to ensure that the classification task was nontrivial.

Two medical student annotators read through over 350 posts on the aforementioned forums and annotated posts as containing misinformation or not. This analysis was performed to determine that a sufficient number of posts contained misinformation in r/essentialoils and r/tanning to establish those forums as misinformation in our data set. To annotate the posts, the annotators used UpToDate and PubMed. UpToDate is the most frequently utilized clinical decision database for physicians, and all information included is evidence-based and peer reviewed [15]. PubMed (the database of science journals for the National Library of Medicine of the National Institutes of Health) supplemented with additional journal articles when needed.

As mentioned, during bot development, we trained the bot to treat all comments related to “tanning” or “essential oils” as positive for misinformation. As a result, we did not exclude posts with phrases such as “avoid tanning,” despite the risk of causing the bot to respond to posts containing accurate information. This workflow was chosen because we felt that false positives were acceptable, but false negatives (where misinformation is present and we failed to reply to it) could be harmful. After the bot had been trained to identify “misinformation” versus “valid posts,” our human annotators reviewed posts to ascertain the number of false positives vs false negatives, using the aforementioned annotation. In our training data set, the percentage of false positives for EOs and tanning was 2% and 5% respectively.

Once the quality of these data sets was verified, we were then able to posit an “accuracy” score for each model to determine how much true misinformation they could assimilate. These scores were calculated by evaluating the trained models on a held-out test set.

Given a smaller proportion of positive training instances (21% and 5% for EOs and tanning, respectively), we created a balanced data set by undersampling the negative examples. We performed a train-test split on this balanced data set (details about their sizes are shown in Table 1).

**Table 1:** Number of instances in the data set.

	Essential oils	Tanning
Training instances, n	1971	586
Test instances, n	221	66

In this study, we aimed to examine the theoretical ability of bots to detect and respond to misinformation. In developing our methods, we found that by using natural language processing techniques, bots can learn differentiating terms such as “tanning,” “essential oils,” or “sun exposure.” These bots have the ability to post prefabricated responses to comments related to a variety of skin conditions. These responses were developed and condensed from the American Academy of Dermatology (AAD) into user-friendly lengths and include a link for viewers to directly access the AAD website.

When these terms are identified, bots can reflexively provide condensed AAD recommendations in a comment. For example, with a mention of sun exposure, the bot can post a brief response detailing risk factors such as blistering sunburns, rates of skin cancer in the United States, and recommendations on sunscreen use. For EOs, the bot can return guidance on safe usage and potential adverse reactions. To be clear, these responses have not been posted in any live forums on Reddit, but the design was aimed at a live endpoint in the future.

### Model Creation

We compared the test accuracy for 3 different models. The first model included a baseline logistic regression model, which used a simple bag-of-words representation considering unigram, bigram, and trigram features. A vocabulary consisting of the 20,000 most frequent ngrams was chosen after converting the text to lowercase.

The second model involved fine-tuning a pretrained Bidirectional Encoder Representations from Transformers (BERT) model [16] with a fully connected feed forward classification layer on top. The posts in the training data were first tokenized using a word piece tokenizer, following which [CLS] and [SEP] tokens were appended to the beginning and end of the sequences, respectively. Adam optimizer with a learning rate of 2e-5 and Binary Cross Entropy Loss was used to finetune this model over 4 epochs.

For the third model, we developed a fine-tuned XLNet model [17] with a single feed forward layer on top for classification. The optimizer, loss, and model hyperparameters were similar to those selected for the BERT model. The held-out test data set was used to evaluate each model’s performance and estimate the prediction error.

## Results

Test accuracies by model are shown in Table 2. We compared the results against a random baseline (where there is an equal probability for each label to be picked for a test instance). Our preliminary results show that all 3 models had high test accuracies for both EOs and tanning. The baseline logistic regression model performed well with an accuracy of over 95%. The top positive features of the logistic regression model included words such as “diffuser” and “blends” for essential oils, “bronzer” and “St. Tropez” for tanning, and top negative features included words such as “rash” and “acne.” The XLNet fine-tuned model was also effective, with a test accuracy over 98%, while the BERT fine-tuned model had the highest test accuracy of 100%.

**Table 2:** Validation accuracy of the models.

Model	Test accuracy for “essential oils,” %	Test accuracy for “tanning,” %
Random predictor	50.00	50.00
Logistic regression model	97.29	95.65
BERT^a^ fine-tuned	99.56	100
XLNet fine-tuned	98.70	98.61

^a^BERT: Bidirectional Encoder Representations from Transformers.

## Discussion

### Principal Findings

Our study demonstrates, in a test environment, the ability for artificially intelligent bots to identify health misinformation related to tanning and EOs on Reddit forums, which have the ability to subsequently post corrective prefabricated responses. These results raise the question of whether benevolent bots should play a role in identifying and intercepting health misinformation on live forums. To date, social media bots have largely failed to promote credible sources. An analysis of 14 million Twitter messages by Shao et al [18] in 2017 revealed that social media bots overwhelmingly spread information from low-credibility sources. They reported that bots can “tailor misinformation” to “target those who are most likely to believe it.” The public’s vulnerability to misinformation is further enhanced by inundation of such untruths from multiple sources. For instance, similar tweets, news stories, and Facebook articles popping up on social media feeds, even if all incorrect, may appear to falsely validate each other [19]. By automatically targeting inaccuracies with accurate medical information, we can potentially interrupt this inundation of untruths.

Beyond the issue of noncredible bots, media coverage related to bots has focused on their potential negative impact on society. These concerns mainly revolve around the use of malicious bots to alter outcomes of elections, seed political and social turmoil, or even endanger lives via public health propaganda. One recent study showed, for example, that bots and Russian trolls on Twitter post more content about vaccination than the average user [20]. However, we would argue that the potential upside makes benevolent bots, at the very least, worthy of further study, with any potential impacts carefully studied before transitioning from proof of concept to real-world application.

Specifically, while bots have been used to spread misinformation, they can also be harnessed proactively to disseminate information from high-credibility sources, such as the National Institutes of Health and various academies of medicine. Indeed, some nonmedical projects have already attempted to harness the power of benevolent bots. For example, the United States Geological Service uses @earthquakeBot, a bot that detects earthquakes of 5.0 magnitude and automatically alerts the public. In 2017, the World Economic Forum experimented with an official Twitter bot, @forumfactbot, to combat misinformation about its funding sources, targeting World Economic Forum–related misinformation in tweets and automatically linking to accurate stories [21]. These examples show how transparent, fact-based bots have previously been harnessed to combat misinformation on social media. The creators of any benevolent bot must preemptively consider all ethical and practical issues prior to and during implementation.

Though concerns about bots are justified, our study builds on a growing body of work arguing that bots can—and should—be studied as forces for public health benefits. Many believe that a critical part of combating misinformation is the strong assertion of the truth, with many effective (though nonbot) examples such as Politifact, Factcheck.org, and Snopes [19]. Others have suggested that the public health community should “go on the offense with our messages,” and perhaps benevolent bots could be 1 avenue to deliver such messages [22]. This reveals the possibility that those with malicious intent could use bots to further their own interests or stymie healthy discussions of differing viewpoints.

### Strengths and Limitations

Our approach has several limitations. Methodologically, we chose to have the bot treat all posts on the r/essentialoils and r/tanning subreddit forums as misinformation. The basis for this assumption came from having annotators read through over 350 posts on r/tanning and r/essentialoils and determine that a sufficient number of posts contained misinformation, which we would be able to consider it misinformation in our data set. Of note, an additional limitation is that while the annotators used evidence-based sources and support from a senior physician to annotate the posts for misinformation, there was no formal training prior to the annotation process. Thus, no standards were developed from which a formal training process could be created.

Many posts simply promoted the practice of tanning, which is undoubtedly misinformation given consensus among experts regarding the risk of melanoma with tanning. Similarly, many posts promoted EO use instead of evidence-based medical treatments. For r/Dermatology, more information was deemed accurate and thus required a different strategy. We considered only posts containing those keywords included in this study on r/Dermatology as misinformation.

Given that bots consider entire forums as misinformation, they are highly sensitive but fairly nonspecific. We run the risk of automatically posting replies to posts containing phrases such as “avoid tanning.” This reflexivity could prompt users to consider the bots as unreliable and thus begin to ignore the responses. In future iterations, we intend to refine this approach to increase the specificity of posts captured.

The bots currently only search for a limited set of keywords, as shown in Multimedia Appendix 1 [23-26]. Given that these keywords do not encompass all the words that users describe when discussing tanning or essential oils, we are inevitably missing posts containing misinformation. We hope to increase the effectiveness of the bots by including a wider set of keywords in future searches, such as commonly used words for tanning in countries outside of the United States.

Furthermore, to be an effective public health intervention, we must assume that users will read both the post containing the misinformation and any corrective responses. However, the massive amount of content on these forums makes it impossible for a casual browser to read everything. Many posts on Reddit are either unread or only have 1 or 2 comments in response to them. The forums are constantly refreshed as new content is generated, meaning that our responses to a post could be buried under a new post within a few hours. One safeguard against this is the “search” function that exists within the forums; if a user is searching for advice on a topic such as “tanning,” Reddit returns results spanning back to the creation of the forum, which could be years prior. The user can then see all posts about the topic, including those that have our responses attached to them.

Another factor complicating Reddit visibility of posts is the order in which posts are displayed. Responses to Reddit posts are displayed in order of how often they are “upvoted,” which is essentially the same function as a “like” on Facebook. Thus, a highly upvoted but inaccurate opinion could become the top comment users see, lending it more visibility than our post. Future research could benefit from addressing how to boost the visibility of validated information, such as running advertisements or “featured spots” on social media sites.

A final limitation is that even if users see factual evidence opposing misinformation, they may disregard corrective responses. Politics and sociology have repeatedly demonstrated that when facts are incongruent with a person’s opinion, a person may in fact disregard the facts presented to them and cling to the misinformation, a phenomenon called “cognitive consistency” [27,28]. Ideological beliefs, or simply rumors heard enough to have reached a “social consensus,” can impair one’s ability to assess the validity of a statement and lead readers to process incongruent information less fluently [27,28]. Further, users may not be receptive to corrective information provided by nonhuman users; indeed, the presence of bots could potentially interrupt a tacit community standard and violate users’ trust, even if bots were completely transparent in their roles to correct health misinformation.

### Conclusions

In our study, our bot models all had high test accuracies, which suggests that artificially intelligent bots may accurately target Reddit posts containing commonly misunderstood health content. The ability to consistently detect comments at risk of misinformation is merely the first step toward using benevolent bots to disseminate high-quality scientific information to the public. Our ultimate goal is to test a novel method of addressing dermatology misinformation on Reddit by posting active replies with bots to posts deemed misinformation. Our results suggest that using artificial intelligence is a potentially beneficial and valid method of targeting misinformation on the internet. Having now established feasibility of both detecting misinformation and reflexively responding to it in test environments, subsequent steps include testing the bots on Reddit and other social media forums, with user satisfaction surveys and links to track user engagement with bot-delivered posts. While this initial work has focused on a subset of dermatology misinformation, it demonstrates proof of concept of the potential for using bots to promote fact-based discussions on any medical topic or public health conversation. Thus, we anticipate continued and necessary work to explore and validate the potential for benevolent bots in the health misinformation space.

## Appendix

Multimedia Appendix 1 Bot responses to misinformation.

## Abbreviations

AAD: American Academy of Dermatology
API: application programming interface
BERT: Bidirectional Encoder Representations from Transformers
EO: essential oil
