# Supplementary material for: Identifying and Responding to Health Misinformation on Reddit Dermatology Forums With Artificially Intelligent Bots Using Natural Language Processing: Design and Evaluation Study
Source: JMIR Dermatol. 2021 Sep 30;4(2):e20975. doi: 10.2196/20975 (PMC10334965; doi:10.2196/20975)
Supplement: Multimedia Appendix 1 [file derma_v4i2e20975_app1.docx]

**Appendix**

Bot response to “essential oils”:

*Please make sure to be careful when using essential oils. They are not regulated by the FDA, meaning manufacturers do not have to prove a product is safe before they sell it. Common reactions to essential oils include: contact dermatitis, allergic reactions, and skin irritation. [23] Lavender oil application has been linked to the development of breasts in young men, [24] and tea tree oil can cause altered mental status when accidentally ingested. [25,26]*

Bot response to sun exposure/tanning:

*According to the American Academy of Dermatology (AAD), skin cancer is the most common cancer in the United States, with 9,500 people per day diagnosed. It can affect anyone, of any race, and the vast majority of cases are due to ultraviolet light exposure, such as that received when tanning. In fact, a single blistering sunburn can double one’s chances of developing melanoma, and 5+ bad sunburns can increase risk by 80%. The AAD recommends avoiding indoor tanning beds and protecting skin with protective clothing, shade, and sunscreen with an SPF of 30 or higher. Click here for more information and tips on how to reduce the risk of getting melanoma. Link: https://www.aad.org/media/stats-skin-cancer*
